# The politics of processed foods: Consumer perceptions of policies targeting ultra-processed foods

**DOI:** 10.1371/journal.pone.0350271

**Published:** 2026-06-01

**Authors:** Brenna Ellison, Maria Kalaitzandonakes, Karen Byrd, Bhagyashree Katare

**Affiliations:** 1 Department of Agricultural Economics, Purdue University, West Lafayette, Indiana, United States of America; 2 Department of Agricultural and Consumer Economics, University of Illinois, Urbana, Illinois, United States of America; University of Modena and Reggio Emilia: Universita degli Studi di Modena e Reggio Emilia, ITALY

## Abstract

This paper examined U.S. public support for six potential government policies designed to regulate ultra-processed foods (UPFs). Specifically, we consider support for two information-based policies (defining UPFs and providing dietary recommendations around UPFs); three restriction-based policies (UPF restrictions in grocery stores, schools, and food assistance programs); and one price-based policy (taxing UPFs). Survey results from 990 U.S. consumers showed the highest support for information-based policies, with the least support for UPF taxation. Additionally, there was considerable heterogeneity in policy support based on perceptions of UPFs, subjective knowledge, and demographics. These results may help policymakers gauge public sentiment around and appetite for regulating UPFs in the U.S.

## Introduction

Nutrition research and advice on food has focused primarily on nutrient intake (e.g., reduced sugar, sodium, saturated fat), rather than the level of processing. The concept of ultra-processed foods (UPFs) initially appeared in the scientific literature in 2010 when Brazilian researchers introduced their novel food classification, NOVA, which groups foods based on “the extent and purpose of industrial processing applied to them” [1]. Recently, this food category has garnered worldwide attention due to research associating UPFs with adverse health outcomes [2]. Based on this evidence, some countries have begun implementing policies aimed at identifying or restricting UPFs [3,4].

The literature has highlighted common types of policy actions relating to UPFs that have been taken thus far across the globe, including establishing dietary guidelines around UPFs, restricting UPFs in various settings (schools, nutrition programs, general food procurement), labeling, taxation, and others [5]. More broadly, policies aimed at reducing consumption of a particular food type often focus on making the product more expensive, providing consumers with information about the product, or restricting where the product can be sold. Research has evaluated policy debates on UPFs across the globe [6]. Authors found that policy advocates tend to highlight implications for health, including the cost of healthcare, and protecting vulnerable consumers such as children, whereas policy opponents tend to stress economic implications (in particular for the food industry) and the implications for governance (i.e., rejection of paternalism).

In the U.S., government intervention relating to UPFs has, thus far, been limited. Pomeranz et al. [7] reviewed a swath of state and federal documents, including bills, proposed rules, and regulations, between 1980 and 2022, and underscored that, while discussions of nutrition and health have been of continued interest, debates on processing or UPFs specifically are quite new. However, the Make America Healthy Again (MAHA) campaign and its affiliated Commission have brought new scrutiny to UPFs in the U.S. The MAHA Commission, which was charged with providing recommendations to the federal administration to address childhood chronic disease (see Executive Order 14212; [8]), highlighted UPFs as an important contributing factor.

In the U.S., Balagtas and Bryant [9] recently found that nearly all consumers were aware of UPFs and a majority expressed moderate levels of concern about UPFs. A smaller proportion of U.S. consumers (about 30 percent) believed that all UPFs should be avoided [9]. Outside of the U.S., Swiss [10], Italian [11], and Brazilian [12] consumers generally associated more processing with less healthy food. However, some Brazilian consumers had positive perceptions of “industrialized food” due to its convenience [12]. In the Netherlands, most Dutch consumers had a slightly negative attitude toward “industrial food processing,” acknowledging both positives and negatives of these foods [13]. In the UK, slightly more than half of respondents reported considering whether a food was ultra-processed in their food decisions [14].

While there have been several evaluations of global consumer sentiment related to UPFs, few studies have evaluated support for policies targeting UPFs. Public sentiment can be an important driver of political action and is thus important to measure. While U.S. food policy has been decided primarily in farm bill discussions in recent years, much of the current efforts around UPFs are being championed by elected officials (e.g., state legislators), underscoring the importance of public sentiment even further. In the case of UPFs, Barrett et al. [15] found that Australian consumers were generally supportive of a label to identify UPFs but were skeptical about its effectiveness and feasibility.

Outside of UPFs, research evaluating public support for food policies (e.g., policies aimed at reducing obesity, sugar-sweetened beverage intake, children’s access to highly caffeinated beverages) has shown that the public is more supportive of information policies and less supportive of price-based policies (e.g., [16–24]).‌‌ Additionally, support for restriction-based policies tends to be lower than support for information-based policies; however, when restrictions focus on children, support tends to increase [20]. Support for food policies often differs across groups, including gender, education, parental status, and political party. Research has also underscored that while differences in food policy support vary across U.S. political parties, more recent increases in support for authoritative approaches by Republicans have narrowed this gap [25].

UPFs may also be considered a food technology, as they are often created using food processing technologies such as emulsifiers, humectants, and so on [26]. From this lens, research has identified that public perceptions of food technologies are a function of their perceived risks and benefits, knowledge, and their attributes and values [27]. Perceptions of risks and benefits of UPFs, along with knowledge about UPFs, may also influence the public’s support or opposition to policies targeting UPFs.

In this paper, we extend the literature to evaluate heterogeneity in U.S. public support for six government policies designed to regulate UPFs. Specifically, we consider two information-based policies (defining UPFs and providing dietary recommendations around UPFs), three restriction-based policies (restrictions on UPFs in grocery stores, schools, or food assistance programs), and one price-based policy (taxing UPFs). We assess variation in public support for each policy across perceptions of risks and benefits associated with UPFs, knowledge of UPFs, and demographic characteristics.

We find that respondents were most supportive of informational policies, with more than 80% saying the U.S. government should formally define UPFs and should provide dietary recommendations on the consumption of UPFs. The majority of respondents were also supportive of restriction-based policies, with higher levels of support for restricting the sale of UPFs in schools compared to restricting the sale of UPFs in grocery stores or limiting their purchases through food assistance programs. Respondents were least supportive of taxing UPF purchases. We find that support for policies was related to perceptions of UPFs, such that perceptions of UPFs being either unsafe or addictive increased support for policy intervention. Perceptions of UPFs being tasty decreased support for some policies, including taxing UPFs and restricting the sale of UPFs. Subjective knowledge (i.e., a respondent’s confidence in their knowledge of UPFs) also increased their support for most policies. Finally, we find support for policies varied across demographics, including income, parental status, education, and political orientation. For example, we find that respondents with lower incomes were less likely to support UPF taxes or restrictions in food assistance programs. Similarly, while some policies did not exhibit differences across politics (e.g., restricting UPFs in schools), others were more supported by Republicans (e.g., restrictions to food assistance programs) or Democrats (e.g., defining UPFs). These results are likely to have important implications for policymakers and other stakeholders currently navigating the evolving UPF policy landscape in the U.S.

## Data and methods

### Participant recruitment

We partnered with Qualtrics panels to recruit U.S. consumers (*n* = 1,023) to participate in an online survey about food and agricultural policy issues, including UPFs. Recruitment took place from February 3 to February 12, 2025. Quota sampling was used to target a nationally representative sample, stratified by gender, age, income, and geographic region. After removing incomplete observations (*n* = 33), we had a final analytical sample of 990 responses. Table 1 summarizes the sample’s demographic characteristics. Our sample was representative of the U.S. population in terms of age; however, our sample over-represented low-income households, households with a college education, and households without children. The study was approved by the Institutional Review Board at the University of Illinois Urbana-Champaign (IRB24-0165).

**Table 1:** Sociodemographic Characteristics of Sample (*n* = 990).

Variable	Sample Proportion (n)	U.S. Population Proportion
*Age*		
18–34 years	0.302 (n = 299)	0.304
34–54 years	0.340 (n = 337)	0.339
55 years and older	0.358 (n = 354)	0.358
*Income* (*annual*)*		
Less than $50,000	0.411 (n = 407)	0.340
$50,000 - $99,999	0.336 (n = 333)	0.290
$100,000 or more	0.253 (n = 250)	0.370
Education: Bachelor’s Degree or Higher*		
Yes	0.386 (n = 382)	0.348
No	0.614 (n = 608)	0.652
Children in Household*		
Yes	0.308 (n = 305)	0.393
No	0.692 (n = 685)	0.607
*Food Assistance Recipient*		
Yes	0.282 (n = 279)	n/a
No	0.718 (n = 711)	
*Political Party*		
Republican	0.384 (n = 380)	n/a
Democrat	0.350 (n = 346)	
Independent/Other	0.267 (n = 264)	

Notes: Proportions may not add to 1 due to rounding. U.S. Population Proportions derived from data from the U.S. Census Bureau. N/a = not available. *Indicates a significant difference (p < 0.05) between the sample distribution and the population distribution based on a Chi-square goodness-of-fit test.

### Survey design

To assess public support for UPF-related policies, respondents were asked whether they believed the government should engage in six potential ways to regulate UPFs. For each potential policy option, respondents could indicate their support with a yes or no response. The six policy options included two information-based policies, three restriction-based policies, and one price-based policy. For information-based policy options, respondents were asked whether the government should (1) formally define UPFs and (2) provide dietary recommendations on the consumption of UPFs. For restriction-based policies, respondents were asked whether the government should restrict (1) the sale of UPFs in grocery stores, (2) the sale of UPFs in schools, and (3) UPF purchases in food assistance programs, such as Supplemental Nutrition Assistance Program (SNAP) and Special Supplemental Nutrition Program for Women, Infants, and Children (WIC). For the price-based policy, respondents were asked whether the government should tax purchases of UPFs. The order of policies presented to the respondents was randomized to prevent ordering effects. Policies were chosen based on the UPF literature and current U.S. policy debate (e.g., [5]). For example, the recent MAHA Commission [28] report explicitly criticized UPFs not being included in dietary guidelines and highlighted concerns with UPFs in schools and federal assistance programs. Further, the Food and Drug Administration and U.S. Department of Agriculture published a request for information for input on developing a uniform definition of UPFs [29].

In line with the literature, we assessed respondents’ perceptions of benefits and risks, knowledge, and personal attributes (e.g., [27]). To measure perceptions of risks and benefits, respondents were asked to rate the extent to which they considered UPFs to be unhealthy/healthy, cheap/expensive, unsafe/safe, unnatural/natural, inconvenient/convenient, not tasty/tasty, and not addictive/addictive. Respondents were asked to rate each attribute pair on a 5-point bipolar scale. The order of attributes was randomized to prevent ordering effects. Table 2 presents the mean scores (±SD) for consumers’ perceptions around health, price, safety, naturalness, convenience, taste, and addictiveness of UPFs.

**Table 2:** Respondent Perceptions of Ultra-Processed Foods (UPFs).

*Attribute Pair*	Mean±SD^a^
*Perceived Risks of UPFs*	
Healthy (1): Unhealthy (5)	3.64 ± 1.34
Safe (1): Unsafe (5)	3.37 ± 1.32
Natural (1): Unnatural (5)	3.67 ± 1.34
Not Addictive (1): Addictive (5)	3.48 ± 1.27
*Perceived Benefits of UPFs*	
Not Tasty (1): Tasty (5)	3.40 ± 1.24
Expensive (1): Cheap (5)	3.24 ± 1.27
Inconvenient (1): Convenient (5)	3.63 ± 1.27

^a^SD = standard deviation.

Respondents perceived UPFs, on average, as tasty and convenient (mean values of 3.40 and 3.63, respectively), yet also unhealthy, unsafe, unnatural, and addictive. UPFs were also perceived as slightly cheaper (mean value of 3.24), though the mean was closest to the scale midpoint value of three. These results align with previous research showing U.S. consumers associated UPFs with risks and benefits [9,30] as well as global consumer perceptions that UPFs were less healthy but more convenient or tasty (e.g., [10–13]).

We would expect consumers who perceive there to be risks associated with UPFs (e.g., unsafe, addictive, unnatural) would be more supportive of policy action, and those who perceive stronger benefits associated with UPFs (e.g., convenient, cheap, tasty) would be less supportive of policies aimed at regulating UPFs. In line with the literature, we expect perceptions of safety and addictiveness to play important roles in consumers’ support for policies. For example, previous research has shown perceptions of food addiction were important in informing perceptions of obesity-related policies (e.g., [31]).

We also measured subjective knowledge (meaning a respondent’s *perceptions* of their own knowledge) as previous research has shown subjective knowledge was important in informing beliefs and decisions around food technology [27]. Additionally, Balagtas and Bryant [9] showed that U.S. consumers displayed a disconnect between subjective and objective knowledge on UPFs, perhaps because UPFs continue to be difficult to define, even for field experts (e.g., [32–35]). Given the lack of a definition of UPFs in the U.S., we do not assess objective knowledge in this study; however, subjective knowledge was measured by asking respondents about their level of confidence in knowing whether a food they see in the grocery store is ultra-processed on a scale from 0 (not confident at all) to 10 (very confident). The average respondent felt somewhat confident in their ability to identify UPFs in the grocery store, with a mean confidence level of 6.0. Confidence varied across consumers; for example, younger consumers and consumers with bachelor’s degrees reported higher levels of confidence (see S1 Table). The impact of UPF subjective knowledge on support for policies is less clear. In general, increased subjective knowledge would suggest the respondent is dialed into the UPF debate and more likely to be supportive of polices; however, expressing a high level of confidence in identifying UPFs may render some policies (e.g., defining UPFs) redundant.

Finally, we included several measures of respondent attributes, including age, income, education, children in the household, food assistance recipient, and political party affiliation, which may impact perceptions of UPF policies. Policies are likely to impact segments differently; namely, people with children in the household would be uniquely impacted by restrictions on UPFs at school, low-income households would be uniquely impacted by a tax on UPFs, and households that utilize food assistance programs would be uniquely impacted by UPF restrictions in such programs. As food policy remains politicized in the U.S., it is also relevant to evaluate differences across political party affiliations. Although political differences in food policy debates remain strong, both blue and red states have taken aim at UPFs or food additives (e.g., [36–38]). We also included age and education as each has been shown to be related to support for food policy issues (e.g., [17]).

### Data analysis

To assess heterogeneity in public support for the six potential government actions relating to UPFs, we modeled respondent *i*’s support for policy *j* as a function of their perceptions of the risks and benefits of UPFs, subjective knowledge on UPFs, and their attributes:


$$\begin{array}{c} {Support}_{ij}=f({UPF\;Perceptions}_{i},{UPF\;Confidence}_{i},\;X_{i}) \end{array}$$ (1)

Here, ${Support}_{ij}$ is equal to 1 if respondent i was in support of the government regulating UPFs using policy option j (1 = define UPFs; 2 = provide dietary recommendations around UPFs; 3 = restrict UPFs in grocery stores; 4 = restrict UPFs in schools; 5 = restrict UPFs on food assistance programs; 6 = tax UPFs) and 0 if they opposed the policy. ${UPF\;Perceptions}_{i}$ is a vector of respondent *i*’s perceptions of UPFs on dimensions of health, price, safety, naturalness, convenience, taste, and addictiveness. ${UPF\;Confidence}_{i}$ is a measure of how confident respondent *i* felt about knowing whether foods are ultra-processed. Finally, $X_{i}$ is a vector of sociodemographic variables that includes age, income, education, children in the household, food assistance recipient, and political party affiliation.

Given there is likely to be correlation across policy preferences (e.g., a respondent who supports defining UPFs may also support providing dietary recommendations around UPF consumption), we used a multivariate probit model to estimate equation 1. The multivariate probit model allows for simultaneous estimation of equation 1 for all six policy options; further, it includes pairwise correlations across the errors of the six equations [39]. As the coefficient estimates from the multivariate probit have limited interpretive value on their own, we also calculated average marginal effects. Data cleaning and descriptive analyses were completed in SAS version 9.4; multivariate probit and average marginal effects estimates were generated in R.

## Results and discussion

**Fig 1:**
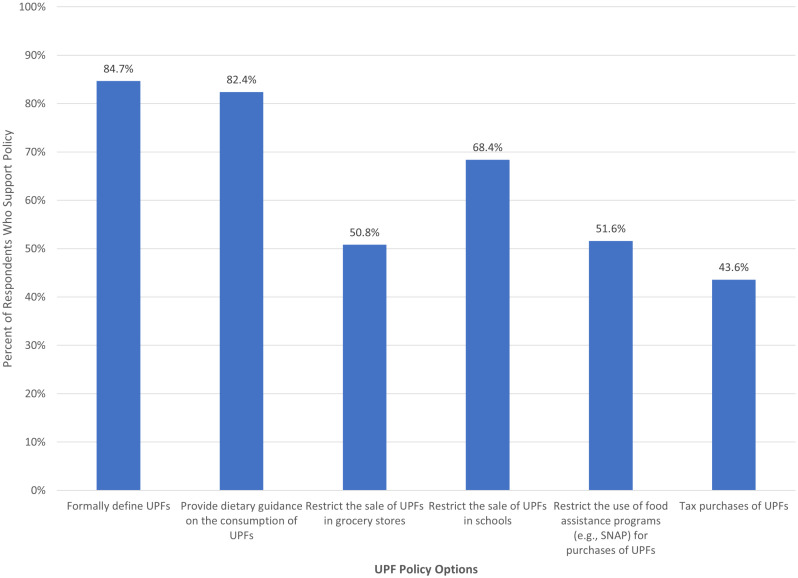
Percent of Respondents Who Supported Policies Targeting Ultra-Processed Foods (UPFs).

Fig 1 presents the proportion of respondents who indicated they supported each policy option. The information-based policies exhibited the highest levels of support, with 84.7% of individuals indicating they would like to see the government formally define UPFs and 82.4% saying they would like the government to provide dietary recommendations around the consumption of UPFs. Restriction-based policies also received majority support, though there was stronger support for restricting the sale of UPFs in schools (68.4% support) relative to grocery stores (50.8% support) or in food assistance programs (51.6% support). Price-based policies failed to garner majority support, with only 43.6% of respondents indicating they would like the government to tax purchases of UPFs. These results align with the literature, which has shown higher levels of support for informational policies (e.g., labeling) and policies aimed at protecting children and lower levels of support for more restrictive policies (e.g., bans) and taxes (e.g., [17,19,20,24]).

S2 Table presents the results from the multivariate probit model. The correlation coefficients at the bottom of the table were all positive and significant, confirming that support across policies was correlated. Correlations were highest between providing dietary guidance on UPFs and defining UPFs and lowest between providing dietary guidance on UPFs and restricting the use of food assistance programs (e.g., SNAP, WIC) for purchases of UPFs.

To improve the interpretability of model results, we report average marginal effects (AMEs) for each variable in Table 3. Columns 1–6 show results for each of the six policies. For factors associated with support for information-based policies (columns 1 and 2), we found that perceptions of safety and addictiveness of UPFs were associated with support for both defining UPFs and providing dietary guidance around UPFs. For example, a one unit increase in *UPF Perception: Unsafe* was associated with approximately a 3 percentage point increase in support for each information-based policy, while a one-unit increase in *UPF Perception: Addictive* was associated with a 2.3 and 2.5 percentage point increase in support for defining UPFs and providing dietary guidance around UPFs, respectively. Perceptions of healthfulness, cost, naturalness, convenience, and taste were not associated with support for either informational policy. Interestingly, while consumers’ confidence in identifying UPFs in grocery stores was not related to support for the government defining UPFs, it was positively related to the government providing dietary guidance around the consumption of UPFs. A one unit increase in confidence was associated with a 1.2 percentage point increase in support for this policy. We also find that younger consumers (18−34 years) were less likely to support information-based policies (AMEs of −10 and −11 percentage points for defining UPFs and providing dietary guidance around UPFs, respectively), and lower-income consumers (household income less than $50,000) were less likely to support providing dietary guidance around UPFs (AME of −9.6 percentage points). Conversely, individuals who identified as Democrats were 7.1 and 7.5 percentage points more likely to support defining UPFs and providing dietary guidance around UPFs, respectively, relative to Independents/Others.

**Table 3:** Average Marginal Effects for Predictors of Support for Policies on Ultra-Processed Foods (UPFs).

Variable	(1) Define UPFs	(2) Provide Dietary Guidance on UPFs	(3) Restrict UPFs in Grocery Stores	(4) Restrict UPFs in Schools	(5) Restrict UPFs in Food Assistance Programs	(6) Tax UPFs
UPF Perception: Unhealthy	0.015 (0.013)	0.016 (0.014)	0.003 (0.018)	0.053*** (0.015)	0.020 (0.019)	0.005 (0.018)
UPF Perception: Unsafe	0.031** (0.012)	0.030** (0.013)	0.058*** (0.017)	0.060*** (0.015)	0.036** (0.018)	0.031* (0.017)
UPF Perception: Unnatural	0.017 (0.014)	0.010 (0.013)	−0.003 (0.017)	0.004 (0.015)	−0.017 (0.020)	−0.030* (0.017)
UPF Perception: Addictive	0.023** (0.010)	0.025** (0.010)	0.039*** (0.013)	0.036*** (0.012)	0.026* (0.013)	0.019 (0.013)
UPF Perception: Tasty	0.007 (0.012)	0.016 (0.012)	−0.045*** (0.015)	−0.025* (0.015)	−0.021 (0.016)	−0.043*** (0.015)
UPF Perception: Cheap	−0.005 (0.012)	0.007 (0.011)	0.008 (0.013)	0.003 (0.012)	−0.007 (0.014)	0.015 (0.013)
UPF Perception: Convenient	0.012 (0.012)	0.005 (0.012)	−0.008 (0.014)	0.029* (0.015)	−0.012 (0.015)	0.001 (0.014)
Confidence Identifying UPFs	0.004 (0.005)	0.012** (0.005)	0.028*** (0.006)	0.024*** (0.006)	0.019*** (0.006)	0.031*** (0.006)
Age: 18–34 years	−0.100*** (0.038)	−0.110*** (0.040)	0.098** (0.043)	−0.066 (0.040)	−0.050 (0.044)	0.086** (0.043)
Age: 35–54 years	−0.058* (0.035)	−0.047 (0.036)	0.100** (0.042)	−0.054 (0.040)	0.031 (0.043)	0.075* (0.042)
Income: Less than $50,0000	−0.059 (0.037)	−0.096** (0.041)	−0.047 (0.045)	−0.019 (0.044)	−0.128*** (0.046)	−0.136*** (0.044)
Income: $50,0000 - $99,999	−0.007 (0.035)	−0.050 (0.040)	−0.060 (0.043)	−0.014 (0.042)	−0.099** (0.043)	−0.076* (0.042)
Bachelor’s degree or higher	0.037 (0.030)	0.039 (0.030)	0.049 (0.036)	0.095*** (0.034)	0.085** (0.037)	0.076** (0.036)
Children in Household	0.036 (0.029)	0.041 (0.030)	0.125*** (0.040)	0.045 (0.036)	0.101** (0.041)	0.110*** (0.041)
Food Assistance Recipient	0.026 (0.029)	0.044 (0.028)	0.094** (0.040)	0.071** (0.033)	−0.004 (0.041)	0.125*** (0.040)
Political Party: Republican	0.021 (0.030)	0.010 (0.031)	0.079** (0.040)	0.021 (0.036)	0.102** (0.041)	0.071* (0.041)
Political Party: Democrat	0.071** (0.028)	0.075** (0.031)	0.091** (0.040)	0.049 (0.037)	0.051 (0.041)	0.095** (0.040)

Notes: Dependent variables were coded as one if respondents said they would support a given policy option and 0 otherwise. Each UPF Perceptions were measured on 5-point scales where 1 = healthy; safe; natural; not addictive; not tasty; expensive; inconvenient and 5 = unhealthy; unsafe; unnatural; addictive; tasty; cheap; convenient. Age categories relative to those 55 years or older. Income categories relative to those with incomes of $100,000 or more. Political party categories relative to Independent/Other. Confidence in identifying UPFs was measured on a 0 = not confident at all to 10 = very confident scale. Standard errors in parentheses. Significance is denoted by *, **, *** for 10%, 5%, and 1% levels, respectively.

For restriction-based policies (columns 3–5), we again found perceptions of safety and addictiveness to be important. Here, both *UPF Perception: Unsafe* and *UPF Perception: Addictive* were significantly associated with support for all three policy options, with AMEs ranging from 3.6 to 6.0 percentage points for *UPF Perception: Unsafe* and 2.6 to 3.9 percentage points for *UPF Perception: Addictive*. The more consumers perceived UPFs as unsafe and addictive, the more likely they were to support the government restricting them in grocery stores, schools, and food assistance programs. Similar to our findings, previous research has shown that food addiction perceptions have been associated with support for obesity-related policies (e.g., [31]). Additionally, subjective knowledge was associated with support for all three restrictions; a one unit increase in confidence in one’s ability to identify UPFs was associated with a 2.8, 2.4, and 1.9 percentage point increase in support for restricting UPFs in grocery stores, schools, and in food assistance programs, respectively.

For restrictions of UPFs at grocery stores (column 3), we also found that consumers who perceived UPFs as tastier (*UPF Perception: Tasty*) were less likely to support this policy (AME of −4.5 percentage points). Conversely, there were many groups who were more likely to support this policy, including younger consumers (AMEs of 9.8 and 10.0 percentage points for 18–34 and 35–54 years, respectively), consumers with children under 18 in their household (AME of 12.5 percentage points), and food assistance recipients (AME of 9.4 percentage points). Additionally, both Republican and Democrat consumers were more likely to support restrictions at grocery stores than individuals who identified as Independent/Other. Identifying as Republican (Democrat) was associated with a 7.9 (9.1) percentage point increase in support for this policy.

In the case of restrictions on UPFs at schools (column 4), we found that in addition to perceptions of safety and addictiveness, perceptions of healthfulness, convenience, and taste were also related to support for this policy. Specifically, consumers who perceived UPFs as being unhealthier (*UPF Perception: Unhealthy*) and more convenient (*UPF Perception: Convenient*) were more likely to support the restriction policy in schools (AMEs of 5.3 and 2.9 percentage points, respectively), while consumers who perceived UPFs as being tastier (*UPF Perception: Tasty*) were less likely to support the policy (AME of −2.5 percentage points). Consumers with a bachelor’s degree and consumers utilizing nutrition assistance programs were 9.5 and 7.1 percentage points more likely, respectively, to support restrictions at schools. Similar to previous research on public support for restricting highly caffeinated beverages at schools, we do not find evidence of differences across political party or parental status [24].

For restrictions of UPFs in food assistance programs (column 5), our results showed that consumers with lower incomes (less than $100,000) were less likely to support this policy (AMEs of −12.8 and −9.9 percentage points for individuals with household incomes less than $50,000 and incomes between $50,000 and $99,999, respectively), while consumers with a college degree and parents were more likely to support this policy (AMEs of 8.5 and 10.1 percentage points, respectively). We also found that Republicans were 10.2 percentage points more likely to support restrictions on UPFs in food assistance programs relative to Independents/Others. While we do not find a significant association between food assistance recipient status and support for this policy, low-income households may be more likely to use or have previous experience with food assistance programs. Research has shown that SNAP recipients are opposed to purchase restrictions as they remove autonomy and do not allow items to be consumed in moderation which would be appropriate for other households [40–42]. It is also worth noting that federal nutrition programs were affected by the One Big Beautiful Bill Act, which was signed into law on July 4, 2025, several months after data collection. This policy option may be viewed differently in light of the changes that have been imposed on these programs.

The price-based policy option (column 6, taxing UPFs) was least popular among consumers overall; yet, there were segments of consumers who were more supportive than others. Consumers who perceived UPFs as unsafe (*UPF Perception: Unsafe*) were more likely to support a tax, and those who perceived UPFs as being tasty (*UPF Perception: Tasty*) were less likely to support a tax. Each unit increase in *UPF Perception: Unsafe* (*UPF Perception: Tasty*) was associated with a 3.1 (4.3) percentage point increase (decrease) in support for this policy. These results align with Miller et al. [21], who found that individuals who believed sugar-sweetened beverages were likely to cause health problems in adults and children were more likely to support a tax on these products. Additionally, consumers with higher levels of confidence in identifying UPFs, younger consumers (18−34 and 35−54 years), consumers with a college degree, parents, and food assistance recipients were more likely to support the policy. Among these groups, AMEs were largest for parents and food assistance recipients; having children in the household was associated with an 11.0 percentage point increase in support while participating in a food assistance program was associated with a 12.5 percentage point increase in support. While both Democrats and Republicans were more likely to support taxing UPFs than Independents/Others (AMEs of 9.5 and 7.1 percentage points, respectively, the effect was only marginally significant for Republicans. As expected, individuals in the lowest income category (less than $50,000) were significantly less likely to support a tax on UPFs (AME of −13.6 percentage points), likely because they would feel the impacts of such a tax more than high-income households.

While we evaluated public support for six relevant policy options, we did not assess all public policy options. For example, we did not evaluate public support for UPF labels, subsidies for minimally processed foods, or restrictions on marketing UPFs. Currently in the U.S., labels relating to UPFs appear eminent, both via state-level regulations requiring labeling of additives or a subset of UPFs and the launch of UPF-free labels from NGOs. Previous research evaluating UPF “warning labels” found no impact on Brazilian consumers’ likelihood of choice when compared to existing front of package labeling [43]. Future research should explore public responses to the different types of expected UPF labels.

Additionally, while evaluations of public perceptions of policies are useful, they are limited in a few important ways [44]. First, as policies considered are hypothetical, evaluations are subject to hypothetical bias, where the public may over- or understate support. Second, evaluations of policies reflect perceptions at a particular time, whereas public perceptions of policies are constantly evolving. As government action and news media coverage evolve, public perception may evolve as well. In the case of this study, data collection occurred before the release of the MAHA Commission initial assessment (May 25, 2025) and subsequent strategy report (September 9, 2025) and the Food and Drug Administration’s request for information (July 25, 2025) to be used as input in developing a definition of UPFs in the U.S. As such, support for policies could be different now given the increased attention to this topic. Third, survey data used to evaluate public policy support may be subject to sampling bias, where respondents’ likelihood of being included in the survey is also related to their likelihood of supporting a policy. For example, our survey overrepresents low-income households, who were more likely to oppose a tax on UPFs or restricting purchases of UPFs in food assistance programs. Fourth, evaluations of public support do not speak to the relative costs or expected effectiveness of public policies. Some studies have shown that price-based policies are more effective than information-based policies at influencing behavior [45–47], while others have found information-based policies or the combination of price and information policies to be more effective [48–50]. Research evaluating the effectiveness of UPF policies is small but growing (e.g., [51,52]).

## Conclusion

There is increasing attention on UPFs in the U.S., with stronger calls, most recently from the MAHA Commission, for government intervention around these products. However, less attention has been given to the public’s perspective of UPF-related policies. Here, we evaluate heterogeneity in public support for a variety of policies related to UPFs.

We found broad support for the government to serve as an information provider on UPFs, with most respondents supporting both providing a formal definition of UPFs and dietary recommendations for UPF consumption. In line with the literature on food policy more broadly, we found lower levels of support for restricting sales of or taxing UPFs, although there appeared to be higher support for restricting the sale of UPFs in schools.

We assessed heterogeneity in support for each policy across respondents’ perceptions of UPFs, finding that consumers who perceived UPFs to be unsafe and addictive were more likely to support regulating UPFs broadly. We also found that perceptions of UPFs being unhealthy increased support for restrictions at schools, and perceptions of UPFs being tasty reduced support for restrictions at grocery stores and taxes. Additionally, consumers who reported higher levels of subjective knowledge were generally more supportive of policies.

We also examined heterogeneity in policy support across consumer attributes, finding that age, income, parental status, and other characteristics were related to policy support. Policies are likely to impact groups differentially across their characteristics. We found that those with lower incomes were less supportive of a tax on UPFs and UPF restrictions in food assistance programs; however, interestingly, we found that both parents and non-parents were equally supportive of restrictions in schools, and those who do and do not utilize food assistance programs were equally supportive of UPF restrictions in the programs. Assessing differences across politics is also particularly relevant, given the rise of MAHA-aligned policies in both red and blue states. Here, we found no political differences in support for restricting UPFs in schools; however, Democrat participants were more likely to be supportive of informational policies, Republican participants were more likely to be supportive of restricting UPFs in food assistance programs, and both were more likely than Independents/Others to be supportive of restricting UPFs in grocery stores and taxing UPFs.

Regulation of UPFs can take many forms and is at various stages in the U.S. and abroad. This paper provides a glimpse into the public’s preferences for regulating UPFs in the U.S. context. As the U.S. moves toward trying to define UPFs and considers related policies targeting UPFs at a national scale, it will be important to monitor how these regulations are communicated and implemented and assess how that ultimately impacts public sentiment around policy efforts.

## Supporting information

S1 Table Associations between Sociodemographic Variables and Confidence in Identifying Ultra-Processed Foods.
(DOCX)

S2 Table Multivariate Probit Coefficient Estimates: Predictors of Support for Policies on Ultra-Processed Foods (UPFs).
(DOCX)

S1 File Dataset. This file contains the raw data used for all analyses.
(CSV)

S2 File Survey Coding File. This file contains the survey questions used and numeric coding for survey questions.
(DOCX)
