# Supplementary material for: The politics of processed foods: Consumer perceptions of policies targeting ultra-processed foods
Source: PLoS One. 2026 Jun 1;21(6):e0350271. doi: 10.1371/journal.pone.0350271 (PMC13225411; doi:10.1371/journal.pone.0350271)
Supplement: S1 Table — Associations between Sociodemographic Variables and Confidence in Identifying Ultra-Processed Foods. (DOCX) [file pone.0350271.s001.docx]

| **S1 Table. Associations between Sociodemographic Variables and Confidence in Identifying Ultra-Processed Foods** | |
| --- | --- |
| Variable | Coefficient Estimate (Standard Error) |
| Age: 18-34 years | 1.038***  (0.212) |
| Age: 35-54 years | 0.872***  (0.204) |
| Income: Less than $50,0000 | -0.217  (0.226) |
| Income: $50,0000 - $99,999 | -0.423*  (0.216) |
| Bachelor’s degree or higher | 0.587***  (0.182) |
| Children in Household | 0.703***  (0.200) |
| Food Assistance Recipient | 0.442**  (0.194) |
| Political Party: Republican | 0.712***  (0.203) |
| Political Party: Democrat | 0.484**  (0.206) |
| Constant | 4.639***  (0.262) |
| *R-square* | 0.106 |
| Notes: Ordinary Least Squares (OLS) Regression used. Confidence in identifying UPFs was measured on a 0=not confident at all to 10=very confident scale. Two responses were excluded from analyses for incomplete demographic information. Age categories relative to those 55 years or older. Income categories relative to those with income of $100,000 or more. Political party categories relative to Independent/Other. Significance is denoted by *, **, *** for 10%, 5%, and 1% levels, respectively. | |
