# Supplementary material for: The politics of processed foods: Consumer perceptions of policies targeting ultra-processed foods
Source: PLoS One. 2026 Jun 1;21(6):e0350271. doi: 10.1371/journal.pone.0350271 (PMC13225411; doi:10.1371/journal.pone.0350271)
Supplement: S2 Table — Multivariate Probit Coefficient Estimates: Predictors of Support for Policies on Ultra-Processed Foods (UPFs). (DOCX) [file pone.0350271.s002.docx]

| **S2 Table. Multivariate Probit Coefficient Estimates: Predictors of Support for Policies on Ultra-Processed Foods (UPFs)** | | | | | | |
| --- | --- | --- | --- | --- | --- | --- |
| Variable | (1)  Define UPFs | (2)  Provide Dietary Guidance on UPFs | (3)  Restrict UPFs in Grocery Stores | (4)  Restrict UPFs in Schools | (5)  Restrict UPFs in Food Assistance Programs | (6)  Tax  UPFs |
| UPF Perception: Unhealthy | 0.070  (0.061) | 0.069  (0.060) | 0.007  (0.051) | 0.169***  (0.049) | 0.053  (0.051) | 0.013  (0.052) |
| UPF Perception: Unsafe | 0.142**  (0.058) | 0.128**  (0.056) | 0.165***  (0.049) | 0.193***  (0.050) | 0.097**  (0.048) | 0.090*  (0.049) |
| UPF Perception: Unnatural | 0.079  (0.063) | 0.043  (0.057) | -0.007  (0.048) | 0.014  (0.049) | -0.046  (0.053) | -0.087*  (0.048) |
| UPF Perception: Addictive | 0.108**  (0.046) | 0.106**  (0.043) | 0.111***  (0.038) | 0.115***  (0.039) | 0.070*  (0.036) | 0.054  (0.037) |
| UPF Perception: Tasty | 0.030  (0.056) | 0.069  (0.053) | -0.127***  (0.043) | -0.080*  (0.048) | -0.057  (0.044) | -0.123***  (0.043) |
| UPF Perception: Cheap | -0.022  (0.054) | 0.028  (0.047) | 0.021  (0.037) | 0.010  (0.040) | -0.018  (0.038) | 0.043  (0.038) |
| UPF Perception: Convenient | 0.055  (0.054) | 0.021  (0.051) | -0.021  (0.040) | 0.092*  (0.047) | -0.032  (0.042) | 0.002  (0.041) |
| Confidence Identifying UPFs | 0.021  (0.025) | 0.053**  (0.023) | 0.079***  (0.018) | 0.076***  (0.021) | 0.052***  (0.018) | 0.090***  (0.018) |
| Age: 18-34 years | -0.431***  (0.157) | -0.438***  (0.150) | 0.277**  (0.123) | -0.206*  (0.125) | -0.134  (0.118) | 0.244**  (0.121) |
| Age: 35-54 years | -0.262*  (0.154) | -0.197  (0.146) | 0.281**  (0.120) | -0.171  (0.124) | 0.083  (0.115) | 0.214*  (0.120) |
| Income: Less than $50,0000 | -0.267  (0.167) | -0.398**  (0.168) | -0.132  (0.126) | -0.061  (0.142) | -0.344***  (0.124) | -0.388***  (0.128) |
| Income: $50,0000 - $99,999 | -0.033  (0.164) | -0.206  (0.162) | -0.171  (0.121) | -0.044  (0.133) | -0.269**  (0.120) | -0.222*  (0.124) |
| Bachelor’s degree or higher | 0.177  (0.143) | 0.169  (0.131) | 0.138  (0.101) | 0.308***  (0.112) | 0.228**  (0.099) | 0.215**  (0.102) |
| Children in Household | 0.172  (0.144) | 0.182  (0.134) | 0.346***  (0.110) | 0.147  (0.117) | 0.271**  (0.109) | 0.304***  (0.112) |
| Food Assistance Recipient | 0.124  (0.141) | 0.194  (0.130) | 0.262**  (0.112) | 0.234**  (0.113) | -0.011  (0.110) | 0.353***  (0.112) |
| Political Party: Republican | 0.101  (0.143) | 0.043  (0.133) | 0.224**  (0.113) | 0.069  (0.118) | 0.276**  (0.111) | 0.203*  (0.117) |
| Political Party: Democrat | 0.349**  (0.147) | 0.336**  (0.145) | 0.256**  (0.115) | 0.157  (0.121) | 0.138  (0.113) | 0.272**  (0.116) |
| Constant | -0.605  (0.389) | -0.782**  (0.399) | -1.430***  (0.314) | -1.878***  (0.355) | -0.574*  (0.313) | -1.016***  (0.321) |
|  |  |  |  |  |  |  |
| *Correlation Coefficients* |  |  |  |  |  |  |
| Define UPFs |  | 0.776*** | 0.414*** | 0.453*** | 0.186** | 0.338*** |
| Dietary Guidance on UPFs |  |  | 0.372*** | 0.413*** | 0.179** | 0.345*** |
| Restrict UPFs in Grocery Stores |  |  |  | 0.774*** | 0.579*** | 0.602*** |
| Restrict UPFs in Schools |  |  |  |  | 0.595*** | 0.634*** |
| Restrict UPFs in Food Assistance Programs |  |  |  |  |  | 0.568*** |
| Notes: Dependent variables were coded as one if respondents said they would support a given policy option and 0 otherwise. Each UPF Perceptions were measured on 5-point scales where 1=healthy; safe; natural; not addictive; not tasty; expensive; inconvenient and 5=unhealthy; unsafe; unnatural; addictive; tasty; cheap; convenient. Age categories relative to those 55 years or older. Income categories relative to those with incomes of $100,000 or more. Political party categories relative to Independent/Other. Confidence in identifying UPFs was measured on a 0=not confident at all to 10=very confident scale. Log-likelihood of the multivariate probit estimation was -2735.6. Standard errors in parentheses. Significance is denoted by *, **, *** for 10%, 5%, and 1% levels, respectively. | | | | | | |
