# Supplementary material for: The politics of processed foods: Consumer perceptions of policies targeting ultra-processed foods
Source: PLoS One. 2026 Jun 1;21(6):e0350271. doi: 10.1371/journal.pone.0350271 (PMC13225411; doi:10.1371/journal.pone.0350271)
Supplement: S2 File — This file contains the survey questions used and numeric coding for survey questions. (DOCX) [file pone.0350271.s004.docx]

Survey Coding File

| 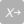 |
| --- |

Gender **What is your gender?**

- Male (1)
- Female (2)
- Non-binary/third gender (3)

| 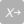 |
| --- |

Age **What is your age in years?**

- Under 18 years (7)
- 18-24 years (1)
- 25-34 years (2)
- 35-44 years (3)
- 45-54 years (4)
- 55-64 years (5)
- 65 or more years (6)

Skip To: End of Survey If What is your age in years? = Under 18 years

| 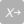 |
| --- |

Income **What is your annual household income before taxes?**

- Under $25,000 per year (1)
- $25,000-$49,999 per year (2)
- $50,000-$74,999 per year (3)
- $75,000-$99,999 per year (4)
- $100,000-$149,999 per year (5)
- $150,000-$199,999 per year (6)
- $200,000 or more per year (7)

| 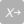 |
| --- |

Region
   **Based on the picture above, in which region do you live?**

- Northeast (1)
- South (2)
- Midwest (3)
- West (4)

Start of Block: UPF

UPF_Terms **To what extent do you think ultra-processed foods are ...**

|  | 1 (1) | 2 (2) | 3 (3) | 4 (4) | 5 (5) |  |
| --- | --- | --- | --- | --- | --- | --- |
| Unhealthy |  |  |  |  |  | Healthy |
| Cheap |  |  |  |  |  | Expensive |
| Unsafe |  |  |  |  |  | Safe |
| Unnatural |  |  |  |  |  | Natural |
| Inconvenient |  |  |  |  |  | Convenient |
| Not Tasty |  |  |  |  |  | Tasty |
| Not Addictive |  |  |  |  |  | Addictive |

| Page Break |  |
| --- | --- |

| 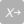 |
| --- |

UPF_Consider **How often do you consider whether something is ultra-processed when purchasing food?**

- Always (1)
- Often (2)
- Sometimes (3)
- Rarely (4)
- Never (5)

UPF_Conf **On a scale from 0, not at all confident, to 10, very confident, how confident do you feel about knowing whether a food you see in the grocery store is ultra-processed?**

|  | Not at all confident | Very confident |
| --- | --- | --- |

|  | 0 | 1 | 2 | 3 | 4 | 5 | 6 | 7 | 8 | 9 | 10 |
| --- | --- | --- | --- | --- | --- | --- | --- | --- | --- | --- | --- |

| Level of confidence () | 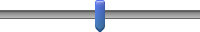 |
| --- | --- |

| 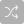 |
| --- |

UPF_Define **Do you think the government should formally define ultra-processed foods?**

- Yes (1)
- No (2)

| Page Break |  |
| --- | --- |

| 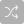 |
| --- |

UPF_DietRec **Do you think the government should provide dietary recommendations on the consumption of ultra-processed foods?**

- Yes (1)
- No (2)

| Page Break |  |
| --- | --- |

UPF_RestrictGroc **Do you think the government should restrict the sale of ultra-processed foods in grocery stores?**

- Yes (1)
- No (2)

| Page Break |  |
| --- | --- |

UPF_RestrictEdu **Do you think the government should restrict the sale of ultra-processed foods in schools?**

- Yes (1)
- No (2)

| Page Break |  |
| --- | --- |

UPF_RestrictSNAP **Do you think the government should restrict the use of food assistance programs (e.g., SNAP, WIC) for purchases of ultra-processed foods?**

- Yes (1)
- No (2)

| Page Break |  |
| --- | --- |

UPF_Tax **Do you think the government should tax purchases of ultra-processed foods?**

- Yes (1)
- No (2)

End of Block: UPF

Start of Block: Demographics

Demo Intro **We'll finish up with some questions about your household.**

| 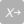 |
| --- |

NumHH **How many people are currently living in your household?**

- 1 person (1)
- 2 people (2)
- 3 people (3)
- 4 people (4)
- 5 people (5)
- 6 people (6)
- 7 people (7)
- 8 or more people (8)

| Page Break |  |
| --- | --- |

Kids **Are there children under 18 years of age currently living in your household?**

- Yes (1)
- No (2)

| Page Break |  |
| --- | --- |

| 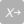 |
| --- |

Edu **What is your current highest level of education?**

- Some High School (1)
- High School Diploma / GED (2)
- Associates or Technical Degree (3)
- Some College (4)
- Bachelor's Degree (5)
- Advanced Degree (MS, MA, PhD, MD, JD, etc) (6)

| 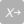 |
| --- |

Hisp **Are you of Hispanic, Latino, or Spanish origin?**

- No, not of Hispanic, Latino, or Spanish origin (1)
- Yes, Mexican, Mexican American, or Chicano (2)
- Yes, Puerto Rican (3)
- Yes, Cuban (4)
- Yes, another Hispanic, Latino, or Spanish origin (5)

Race **What is your race?**

- White (1)
- Black or African American (2)
- American Indian or Alaska Native (3)
- Asian (4)
- Native Hawaiian or Pacific Islander (5)
- More than one race (6)
- Other - please specify: (7) __________________________________________________

| Page Break |  |
| --- | --- |

| 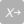 |
| --- |

Metro **Do you live in a large metropolitan area (including suburbs) such as Chicago, Dallas/Fort Worth, New York City, etc?**

- Yes (1)
- No (2)

| Page Break |  |
| --- | --- |

RURAL **What best describes the community you live in?**

- Urban (1)
- Suburban (2)
- Rural (3)

| Page Break |  |
| --- | --- |

FarmFam **Do you or anyone in your immediate family (grandparents, parents, siblings, aunts, or uncles) farm or ranch for a living?**

- Yes (1)
- No (2)

| 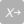 |
| --- |

FoodAssist **Is your household currently participating in any food and nutrition assistance program (SNAP, WIC, School Meals, etc.)?**

- Yes (1)
- No (2)

| Page Break |  |
| --- | --- |

PolViews **In general, would you describe your political views as...**

- Very liberal (1)
- Liberal (2)
- Moderate (3)
- Conservative (4)
- Very conservative (5)

| Page Break |  |
| --- | --- |

| 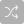 |
| --- |

PolParty **With which political party do you most identify?**

- Republican (1)
- Democratic (2)
- I am an independent (3)
- Other (4) __________________________________________________

| Page Break |  |
| --- | --- |
